# Highly sensitive therapeutic drug monitoring of infliximab in serum by targeted mass spectrometry in comparison to ELISA data

**DOI:** 10.1186/s12014-024-09464-x

**Published:** 2024-02-29

**Authors:** Andreas Hentschel, Gina Piontek, Rob Dahlmann, Peter Findeisen, Roman Sakson, Phil Carbow, Thomas Renné, Yvonne Reinders, Albert Sickmann

**Affiliations:** 1 Leibniz-Institut für Analytische Wissenschaften-ISAS-e.V., Dortmund, Germany; 2 MVZ Labor Dr. Limbach & Kollegen, Heidelberg, Germany; 3 Institute of Clinical Chemistry and Laboratory Medicine, University Medical Center Hamburg-Eppendorf, Hamburg, Germany; 4 Irish Centre for Vascular Biology, School of Pharmacy and Biomolecular Sciences, Royal College of Surgeons in Ireland, Dublin, Ireland; 5 Center for Thrombosis and Hemostasis (CTH), Johannes Gutenberg University Medical Center, Mainz, Germany; 6 Medizinisches Proteom-Center, Ruhr-Universität Bochum, Bochum, Germany; 7 Department of Chemistry, College of Physical Sciences, University of Aberdeen, Aberdeen, United Kingdom

**Keywords:** TDM, Infliximab, Absolute quantification, Mass spectrometry

## Abstract

**Background:**

Presently, antibody concentration measurements for patients undergoing treatment are predominantly determined by ELISA, which still comes with known disadvantages. Therefore, our aim was to establish a targeted mass-spectrometric assay enabling the reproducible absolute quantification of peptides from the hypervariable and interaction regions of infliximab.

**Methods:**

Peptides of infliximab were measured post-trypsin digestion and subsequent separation on a Vanquish Horizon UHPLC coupled to a TSQ Altis Triple-Quad mass spectrometer. Normalization and absolute quantification were conducted using stable isotope-synthesized peptides. Calibration curves covering a range of 0.25-50 µg/ml were employed for quantitation.

**Results:**

We demonstrated the substantial influence of peptide selection, choice of hydrolase for digestion, and digestion time on absolute peptide yield (28–44% for peptide 1 and 64–97% for peptide 2). Furthermore, we showed that the generated calibration curves for absolute quantification were highly reproducible and robust (LLOQ1 0.72 µg/ml and LLOQ2 1.00 µg/ml) over several months. In comparison to ELISA values, the absolute values obtained by mass spectrometry often yielded lower results for both targeted peptides.

**Conclusions:**

In this study, a semi-automated workflow was employed and tested with 8 patients and corresponding replicates (*n* = 3–4). We demonstrated the robust implementation of calibration curves for the absolute quantification of infliximab in patient samples, with coefficients of variation ranging from 0.5 to 9%. Taken together, we have developed a platform enabling the rapid (2 days of sample preparation and 30 min of measurement time per sample) and robust quantification of Infliximab antibody concentration in patients. The use of mass spectrometry also facilitates the straightforward expansion of the method to include additional antibody peptides.

**Supplementary Information:**

The online version contains supplementary material available at 10.1186/s12014-024-09464-x.

## Introduction

Therapeutic Drug Monitoring (TDM) refers to the measurement of drug concentrations in patients’ blood sera or plasma, precisely monitoring these drug levels to assess therapeutic effectiveness and potential toxicity of medications in various medical fields such as oncology, immunology, infectious diseases, and psychiatry [1]. Currently, conventional TDM methods primarily focus on the use of Enzyme-Linked Immunosorbent Assay (ELISA) and other immunological tests to determine drug concentrations in the blood [2, 3]. However, these methods face challenges, including limited sensitivity and specificity, the risk of cross-reactivity with similar molecules, and the time-consuming and costly need to develop and conduct separate tests for each medication [4, 5]. In particular, anti-drug antibodies (ADA) can influence the results of immunological tests, such as ELISA, by blocking respective epitopes. Furthermore, measurement results could be distorted by the presence of ADA or heterophilic anti mouse antibodies (HAMA) that might cross-react with the diagnostic antibodies in the test kits. Such cross-reactivity can lead to false-positive or false-negative results, compromising the accuracy and reliability of TDM analysis [6] As a result, traditional TDM methods heavily reliant on immunological tests may be unreliable and results from different kits might vary substantially [7].

The integration of mass spectrometric methods in the field of TDM represents a promising approach. These techniques are based on the precise detection and quantification of peptide chains and are less susceptible to cross-reactivity with autoantibodies, as they can specifically detect the variable region of the antibodies to be determined.

In the area of inflammatory bowel diseases (IBD), a group of chronic autoimmune disorders affecting the digestive tract, the introduction of monoclonal antibodies (MAbs) targeting TNF-α marked a significant advancement. Notably, infliximab (IFX) and Adalimumab (ADM) revolutionized therapy, significantly improving the quality of life for patients and decrease the need for abdominal surgeries [8].

A focused approach to TDM during the induction phase has proven essential for achieving optimal therapeutic outcomes avoiding loss of response of the respective drug. Maintaining IFX or ADM concentrations above certain thresholds, so called trough level, demonstrated a clear association with improved treatment responses and overall positive outcomes in IBD patients.

Despite the general therapeutic efficiency, over a third of patients responding to TNF-α inhibitors exhibit no response to induction therapy (primary non-responders). Additionally, up to 50% of initial responders experience a decline in therapy effectiveness over time (secondary non-responders) [8, 9].

The reasons for primary nonresponse extend beyond TNF-α and are mediated by disease processes influenced by proinflammatory molecules. A profound understanding of these mechanisms is indispensable for developing individualized therapeutic approaches.

Factors such as reduced albumin levels, high Body Mass Index, systemic inflammation, and immune response to therapy are associated with secondary nonresponse [10]. Similarly, immunogenicity plays a role, underscoring the importance of a detailed investigation of these factors within the framework of TDM.

Laboratory diagnostics for quantifying concentrations of MAbs in the bloodstream and assessing immunogenicity play a pivotal role in optimizing therapeutic approaches, especially when partial treatment responses or treatment failures are observed. Precision in laboratory results yields valuable insights that significantly affect patient management.

Through the application of mass spectrometry, selective and accurate measurements of MAbs in the blood are obtainable, enhancing the reliability and accuracy of TDM analysis and minimizing the effects of autoantibodies on test results. Additionally, mass spectrometry offers the capability of multiplexing, allowing the simultaneous detection and quantification of multiple MAbs in a patient sample, especially pivotal for patients under therapy using a combination of antibodies.

Given these challenges, there is an urgent clinical need for the development of new methods that enable more precise, efficient, cost-effective and adaptable approaches to targeted drug monitoring.

## Methods

### Chemicals and materials

SILu™Lite SigmaMAb infliximab Monoclonal Antibody, Tris(2-carboxyethyl) phosphine hydrochloride (TCEP), and Iodoacetamide (IAA) was purchased from Sigma-Aldrich (St. Louis, US). Trypsin Gold (trypsin 1), Platinum (trypsin 2) and sequencing grade (trypsin 3) were purchased from Promega (Madison, US). Trypsin sequencing grade (trypsin 4) Trypsin MS approved (trypsin 5) was purchased from Serva Electrophoresis (Heidelberg, Germany). Recombinant trypsin (trypsin 6) trypsin bovine (trypsin 7) purchased from Sigma-Aldrich (St. Louis, US). Oasis HLB µElution Plate 30 μm was purchased from Waters (Eschborn, Germany). All solvents used for chromatography were purchased from Biosolve (Biosolve BV, Valkenswaard, Netherlands) with LC-MS-grade quality.

### Patient cohort and sample material

Anonymous leftover serum samples were collected from the MVZ Laboratory Dr. Limbach after routine analysis. The handling of specimens for this laboratory-based analytical study was in accordance to the prerequisites that have been defined by the Central Ethics Committee of the German Medical Association^1^. In particular, the cohort consists of human blood serum from 8 patients who are being treated with the monoclonal antibody infliximab. Samples were aliquoted, snap frozen using liquid nitrogen, and stored at -80 °C until used. In addition, for each serum sample corresponding ELISA measurement values were available.

### Calculation of digest yield of the antibody infliximab peptides

To assess the most suitable hydrolase in terms of digest yield and reproducibility, two distinct experiments were conducted. To ensure a fair comparison of digestion efficiencies among different hydrolases in an exploratory experiment, the same sample matrix (1 µl NIST plasma) with a fixed digestion time of 16 h was used for each analyis. Moreover, the digestion process adhered consistently to the same protocol, maintaining an identical protein:trypsin ratio of 20:1. To determine the digestion yield, 500 µg SILu^TM^Lite SigmaMAb infliximab Monoclonal Antibody (infliximab Standard, Sigma-Aldrich, USA) was first rehydrated and then diluted with plasma as matrix to obtain a final concentration of 0.025 µg/µL antibody in solution. Subsequently, 1.25 µg per vial was diluted according to the conventional FASP protocol to finally obtain a concentration of 3.79 µg/ml after elution of the digest from the FASP filter reflecting complete digestion. To determine the experimental yield of the digest, a stable isotope labeled standard of known concentration was added to the digested peptides. The corresponding peak areas of standard to digested peptide reflect the experimentally determined digestion yield in regard to the theoretical amount per well.

To further explore the results of these experiments, we performed a time-resolved study in a second experiment to investigate the effects of different incubation times on digestion of the antibody. For this purpose we used the best performing trypsin from the exploratory study. Specifically, incubation periods of 2 h, 4 h, 6 h, and 16 h were systematically tested using the same experimental setup described above.

### Comparison of four common sample preparation methods

In conjunction with the digestion yield experiments we compared four sample preparation methods: S-Trap 2-hour digestion (as recommended by the manufacturer), S-Trap 16-hour digestion, FASP and in-solution digestion. Each method involved the processing of five NIST plasma samples, with two individuals working on the preparation independent from each other. In total, each preparation was accomplished in 10 technical replicates from a pooled plasma sample to monitor the reproducibility.

### S-Trap

The buffer used contained 5% SDS and 100 mM triethylammonium bicarbonate (TEAB), PhosStop and Protease Inhibitor. The binding buffer used subsequently, consisting of 50 ml 1 M TEAB, was adjusted to a pH of 7.2. For plasma, 5 µl of the plasma sample was taken and diluted with 95 µl of a freshly prepared SDS lysis buffer. Of this solution, 30 µl were taken for reduction and alkylation. For reduction, 0.6 µl of a 500 mM TCEP solution was added to the sample, and it was incubated for 30 min at 37 °C with shaking. After the incubation period, 0.9 µl of a 500 mM IAA solution was added. Another incubation step was conducted in the dark at room temperature with shaking for 30 min. Subsequently, 3.5 µl of phosphoric acid (12%) and 210 µl binding buffer were added to each sample. Sample clean up and proteolysis were carried out using the S-Trap protocol (Protifi) for S-Trap Mini cartridges using a protein to trypsin ratio of 10:1. Samples were incubated either 2 h at 47 °C or 16 h at 37 °C. After desalting, the samples were dried down and reconstituted at a concentration of 3.3 µg/µl.

### FASP

For the preparation and digestion of plasma according to FASP, the plasma sample underwent alkylation. Therefore, 30 µl of a 50 mM ABC buffer (pH 8.5) and 7 µl of a 10% DOC (sodium deoxycholate) solution were added to 5 µl of the sample, followed by shaking for five seconds. Subsequently, 1 µl of 500 mM TCEP was added, and the mixture was incubated at 37 °C for 30 min. After the incubation period, a 500 mM IAA solution was added until a final concentration of 15 mM was reached. Another incubation step was carried out in the dark at room temperature with shaking for 30 min. The FASP filter was conditioned with 100 µl of the 8 M urea solution and centrifuged at 13,800 rcf for 5 min. The eluate was discarded in the same manner as for the sample loading and washing steps. The alkylated sample was mixed with the 8 M urea solution on the filter and centrifuged at 13,800 rcf for 20 min. Washing steps were performed three times with 100 µl of 8 M urea solution and three times with 100 µl of 50 mM ABC solution. Centrifugation followed each washing step. For each sample, 100 µl digestion buffer (100 µg trypsin in 1 ml 50 mM ammonium bicarbonate (ABC), 2 µl 1 M CaCl_2_ solution) was added (trypsin to substrate ratio of 1:10), treated for 5 min at 37 °C with shaking, and then incubated for 16 h at 37 °C. Subsequently, 50 µl 50 mM ABC were pipetted onto the filter and centrifuged. Finally, 50 µl ultrapure water was added and centrifuged, completing the elution and enabling the disposal of the filter. To achieve a pH value of < 3.0, 20 µl of 10% TFA was added to the sample solution and checked with pH paper. The sample was frozen in this dissolved state at -80 °C.

### In solution digestion

For in-solution digestion, 5 µl of plasma (NIST) or serum (patients) was initially added to 10 µl of the urea buffer in a 96-well plate. Subsequently, 3.6 µl of 50 mM TCEP was added and incubated for 30 min at 37 °C. For alkylation, 1.4 µl of a 250 mM IAA solution was added to the wells, mixed, and allowed to stand for an additional 30 min at room temperature, protected from light. A volume of 6.1 µl of the sample was then transferred to a new reaction vessel. To initiate digestion, 100 µg of trypsin was dissolved in 450 µl of ABC buffer, and 0.9 µl of CaCl_2_ solution was added. Subsequently, 45 µl of each was added to the samples to achieve a final trypsin-to-substrate ratio of 1:10. The mixture was then incubated at 37 °C for approximately 16 h. Digestion was halted by adding 5.1 µl of 10% formic acid (FA). Purification was carried out simultaneously for both the FASP and in-solution samples using the same protocol. All subsequent steps were executed with the semi-automated sample preparation device Resolvex A200 from Tecan (Männedorf, Switzerland). To prepare the 96-well filter plates, they were conditioned twice with 100 µl of ACN with 0.1% TFA and twice with 100 µl of 0.1% TFA in water. The samples were then transferred to the filters. Subsequent washing steps were performed three times with 100 µl of 0.1% TFA, followed by elution twice with 50 µl of 60% ACN containing 0.1% FA. The plate was dried and frozen at -80 °C until further analysis.

### Quality control of prepared samples

Following the desalting process, the entirety of the proteolytic digests underwent meticulous evaluation for complete digestion using monolithic column separation (PepSwift monolithic PS-DVB PL-CAP200-PM, Dionex) integrated into an inert Ultimate 3000 HPLC system (Dionex, Germering, Germany). This assessment involved the direct injection of a 1 µg sample. Utilizing a binary gradient (solvent A: 0.1% TFA, solvent B: 0.08% TFA, 84% ACN) with a progression from 5 to 12% B within 5 min and subsequently from 12 to 50% B over 15 min, the flow rate was maintained at 2.2 µl/min, and the temperature was set at 60 °C. UV traces were recorded at 214 nm throughout this procedure [11].

### Data independent-acquisition LC-MS/MS

All samples were analyzed using an UltiMate 3000 RSLC nano UHPLC coupled to a QExactive HF mass spectrometer, and 1 µg of peptide mix was applied. Samples were first transferred to a 75 μm × 2 cm, 100 Å, C18 precolumn at a flow rate of 10 µl/min for 20 min., followed by separation on the 75 μm × 50 cm, 100 Å, C18 main column with a flow rate of 250 nl/min and a linear gradient consisting of solution A (99.9% water, 0.1% formic acid) and solution B (84% acetonitrile, 15.9% water, 0.1% formic acid), with a pure gradient length of 120 min (3–45% solution B). The gradient was applied as follows: 3% B for 5 min, 3–35% for 120 min, followed by 3 wash steps, each reaching 95% buffer B for 2 min. After the last wash step, the instrument was allowed to equilibrate for 20 min. MS data acquisition was performed in DIA (data independent acquisition) mode using an in-house generated spectral library. Each analyzed sample was mixed with an appropriate amount of iRT standard (Biognosys). Full MS scans were acquired from 300 to 2,000 m/z at a resolution of 60,000 (Orbitrap) using the polysiloxane ion at 445.12002 m/z as the lock mass. The automatic gain control (AGC) was set to 3E6 and the maximum injection time was set to 20 milliseconds. The full MS scans were followed by 23 DIA windows, each covering a range of 28 m/z with an overlap of 1 m/z starting at 400 m/z and acquired at a resolution of 30,000 (Orbitrap) with an AGC of 3E6 and an nCE of 27 (CID). For analysis of samples acquired by nanoscale liquid chromatography coupled to mass spectrometry (LC-MS/MS) in DIA mode, data were imported into Spectronaut software (Biognosys) and analyzed using a library-based search. The search and extraction settings were kept as default (BGS Factory settings). Human proteome data from UniProt (www.uniprot.org) with 20,374 entries were selected as the proteome background. For reliable label-free quantification, only proteins identified with ≥ 2 unique peptides were considered for further analysis. Next, the average normalized abundance (obtained by Spectronaut) for each protein were calculated and used to determine the ratios of patient muscle samples with their respective controls. Finally, a log2 transformation of the generated ratios and Student’s t-test p-values were calculated for each protein using MS Excel.

### Setup and optimizing a targeted assay for infliximab by LC–MS/MS using MRM

**Fig. 1:**
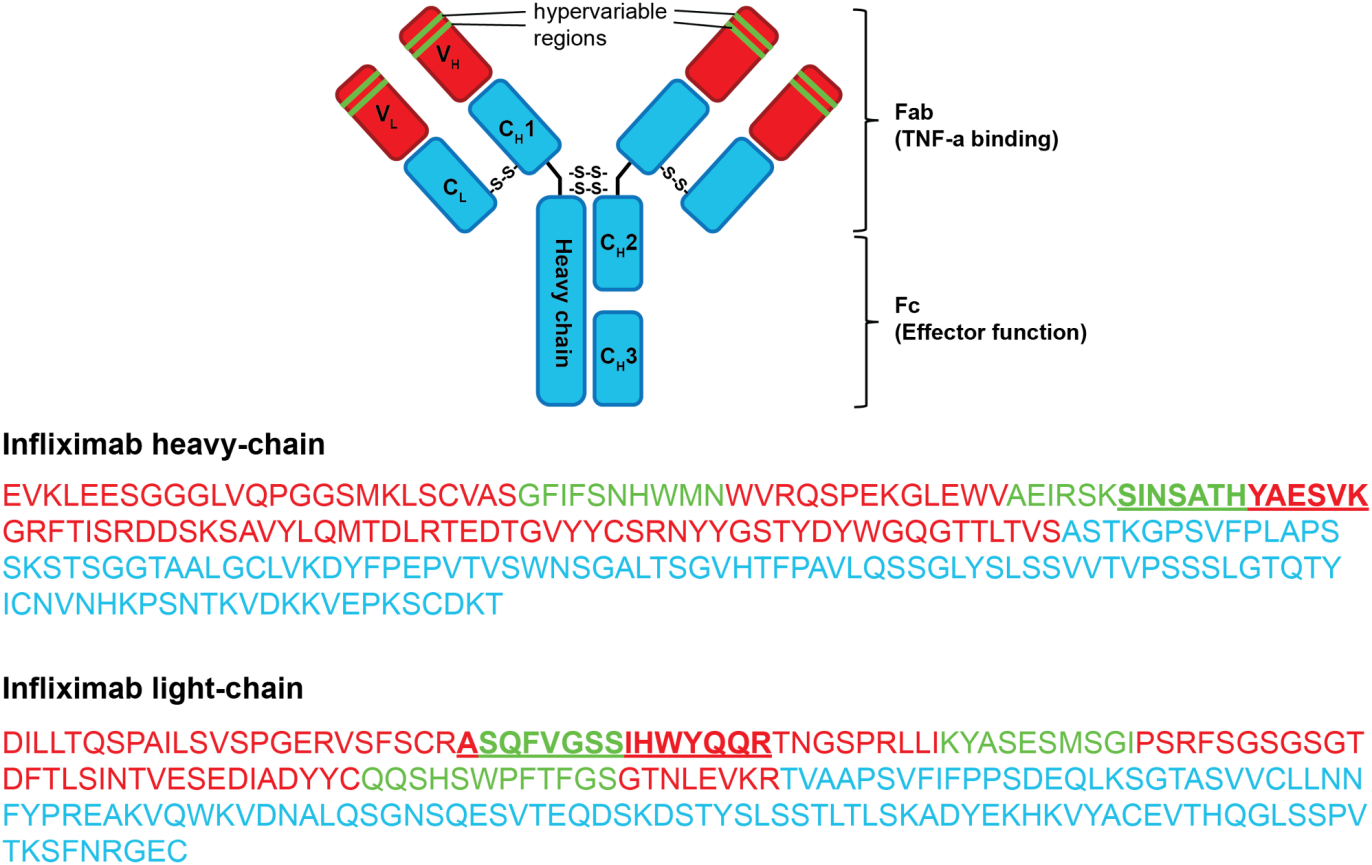
Schematic structure of infliximab and its amino acid sequence depicting Fab heavy and light chains. Blue regions represent conserved peptide sequence, red shows unique amino acid sequences and green the hypervariable regions and interaction points with TNFa. The Peptide sequences selected for quantification are marked with bold and underlined

The peptides suitable for quantification were synthesized in-house by use of ^15^N and ^13^C stable isotope-labeled arginine and lysine (Fig. 1). The selection of peptides was limited to tryptic peptides. In addition, only peptides from the variable range of monoclonal antibodies were selected. This ensured that the peptides subsequently identified by mass spectrometer were antibody-specific. Verification was carried out by localizing the peptide in the amino acid sequence of the antibody. The BLAST algorithm (Basic Local Alignment Search Tool) was used for this purpose [12]. Furthermore, each of the peptides was also available in a natural, non-labeled version for usage as internal standard at a later point.

The fragment ions were selected in the Skyline software using the Prosit neural network architecture [13]. Five fragment ions were determined for each previously selected precursor ion. The preliminary selection of fragment ions was based on the top 5 most intense fragment ions calculated by Prosit in silico. An overview of the selected transitions can be found in Table 1. These fragments were then verified by comparing the spectra calculated by Prosit with experimentally determined spectra. The top 5 most intense fragment ions per precursor ion determined in this way were then saved in Skyline for all further measurements.

All MRM measurements were performed with a Vanquish Horizon UHPLC coupled to a TSQ Altis Triple-Quad mass spectrometer (all Thermo Fisher Scientific, Germany) using a binary reversed phase gradient (Waters Acquity UPLC Peptide CSH C18; 1 mm x 150 mm), equipped with a respective VanGuard column at a flow rate of 100 µL/min (0–18 min, 3–35%, 18–20 min, 35–95%, 20–24 min 95%, 24–25 min 3%, 25–26 min 95%, 26–34 min 3%). First, an equimolar mix of 3.47 pmol/µl was prepared from the SIL peptides in 0.1% TFA. Transitions were monitored in positive ion mode using dynamic MRM acquisition with a detection window of 2.5 min, 3 s cycle time, Q1 resolution (FWHM) of 0.7, Q3 resolution (FWHM) of 1.2, and a dwell time of at least 290 ms. All MRM raw data were processed and inspected using the Skyline software (version 23.1.0.268).

For scheduling, 3.47 pmol per peptide was injected to the HPLC and the retention windows of the fragment ions were determined using the Skyline software [14]. All measurements were carried out in scheduled MRM mode with a retention time window of 2.5 min. The best-responding transitions were monitored by MRM-MS for each peptide in all subsequent assay development experiments. A summary of the developed targeted assay used for quantitation can be found in Table 1.

**Table 1:** Summary of the chosen Infliximab peptides with their corresponding precursor and product m/z values and their optimized collision energy

Peptide sequence	Precursor (m/z)	Product (m/z)	Opt. CE (V)
SINSATHYAESVK(+ 3)	469.568	467.735	5.4
SINSATHYAESVK(+ 3)	469.568	503.254	9.4
SINSATHYAESVK(+ 3)	469.568	546.77	8.4
SINSATHYAESVK(+ 3)	469.568	603.791	7.4
SINSATHYAESVK(+ 3)	469.568	696.356	15.4
SINSATHYAESVK (heavy)(+ 3)	472.239	471.742	5.4
SINSATHYAESVK (heavy)(+ 3)	472.239	507.261	9.4
SINSATHYAESVK (heavy)(+ 3)	472.239	550.777	8.4
SINSATHYAESVK (heavy)(+ 3)	472.239	607.798	7.4
SINSATHYAESVK (heavy)(+ 3)	472.239	704.37	15.4
ASQFVGSSIHWYQQR(+ 3)	598.628	631.307	9.2
ASQFVGSSIHWYQQR(+ 3)	598.628	680.841	11.2
ASQFVGSSIHWYQQR(+ 3)	598.628	754.376	10.2
ASQFVGSSIHWYQQR(+ 3)	598.628	780.379	19.2
ASQFVGSSIHWYQQR(+ 3)	598.628	917.438	20.2
ASQFVGSSIHWYQQR (heavy)(+ 3)	601.964	636.311	9.2
ASQFVGSSIHWYQQR (heavy)(+ 3)	601.964	685.846	11.2
ASQFVGSSIHWYQQR (heavy)(+ 3)	601.964	759.38	10.2
ASQFVGSSIHWYQQR (heavy)(+ 3)	601.964	790.387	19.2
ASQFVGSSIHWYQQR (heavy)(+ 3)	601.964	927.446	20.2

The optimal CE was calculated using Skyline based on the manufacturer’s specifications for the slope and the y-intercept of the respective charge states. For optimization of the CE, collision energy is ramped stepwise (step size 1.0 V). The experimentally determined optimum CE for each fragment ion was selected based on the signal intensities. These were stored in the method and automatically selected by Skyline for all further measurements. Therefore, raw data was imported into Skyline and the quantification of the monoclonal antibody was carried out using a calibration curve calculated by Skyline. To establish the calibration curves, different amounts of SIS peptides range from 0.25 to 50 µg antibody /ml plasma were spiked into 10 µg of tryptic digested serum as the matrix for the calibration curves in the experiment with addition of corresponding unlabeled peptides with a concentration of 12.5 µg antibody /ml for normalization. Technical triplicates of the individual samples were injected in order of lowest to highest concentration. The calibration line was created using the ratio of the area under the curve of native to isotope-labeled peptides and analyzed without weighting to determine the assay’s linear range and LLOQ. Peptide Settings for quantification and figures of merit were set here as followed: linear regression fit, no regression weighting, maximum LOQ bias 20%, maximum LOQ CV 20%, LOD calculated on blank plus 3*standard deviation.

### Semi-automated sample processing and trypsin digestion

**Fig. 2:**
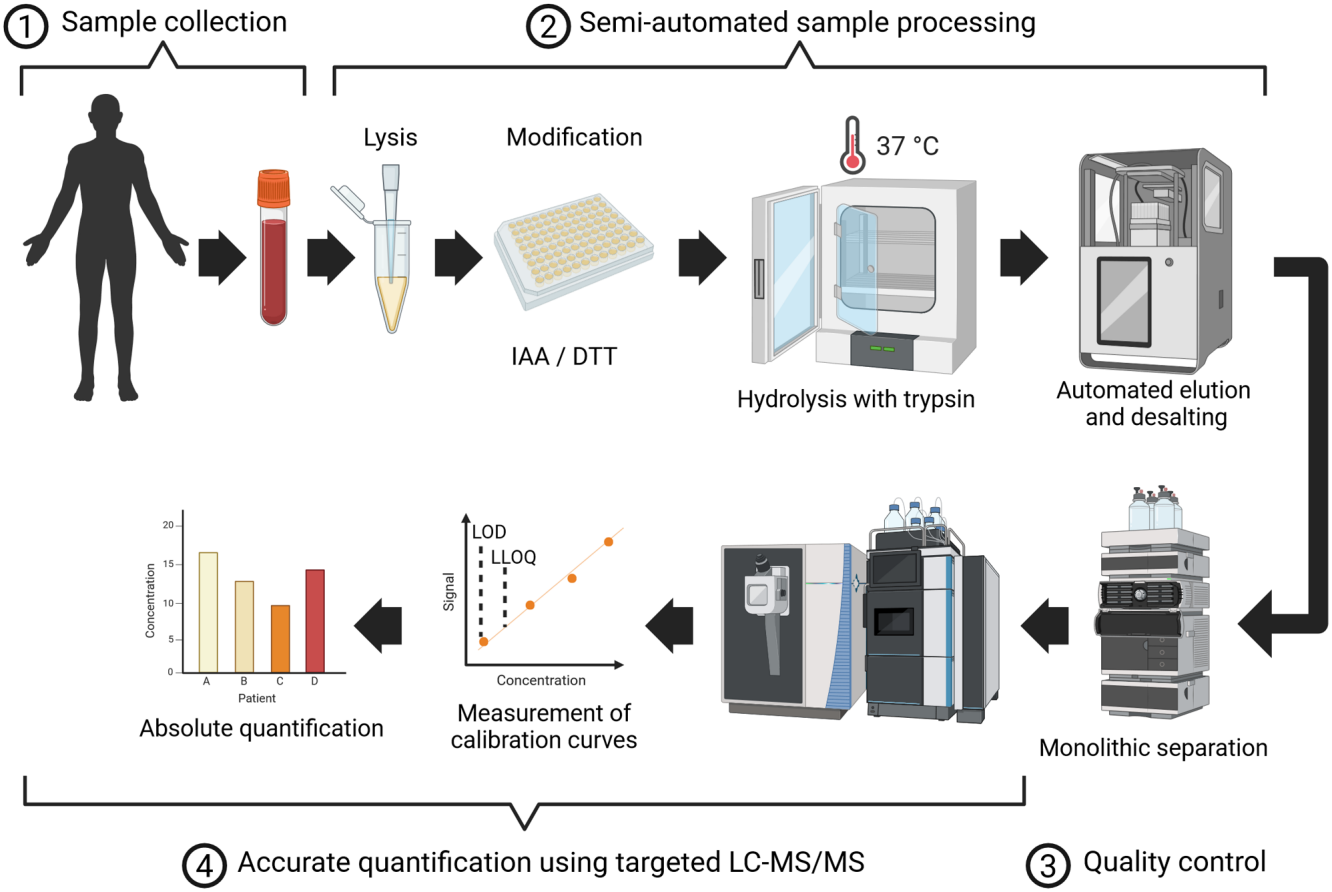
Semi-automated proteomic workflow: To enhance reproducibility in the sample preparation of TDM samples, an automated 96-well plate FASP process was employed. This allowed for the concurrent preparation of over 90 samples, minimizing manual pipetting steps to reduce inaccuracies and save time. (Figure was created using BioRender)

Finally, 5 µL patient serum were added to 10 µL 9 M urea and reduced using 10 mM TCEP for 30 min at 37 °C and further alkylated in 30 mM of IAA for 30 min at room temperature in the dark. The remaining IAA was quenched with 20 mM dithiothreitol (DTT) for a further 15 min at room temperature. Subsequently, samples were digested using trypsin (Serva, ratio [protease:protein] 1:10 in 50 ammonium bicarbonate buffer supplemented with 2 mM CaCl_2_) at 37 °C for 2 h according to our results of the time-resolved antibody digestion experiment. Digestion was stopped by acidification using 10% formic acid. Finally, peptides were de-salted in a semi-automated way using a Waters OASIS HLB µElution plate, ending up after lyophilisation and re-constitution of the peptides in 95.7 µL 0.1% trifluoroacetic acid (TFA). A comprehensive overview of the final semi-automated workflow for Infliximab quantitation in human serum samples is shown in Fig. 2.

## Results

### Optimizing matrix sample preparation

**Fig. 3:**
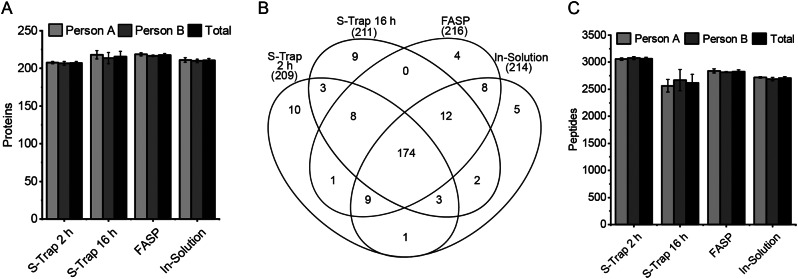
Comparison of different sample preparation methods: (**A**) Comparison of identified proteins with high significance measured in DIA mode for four different sample preparation (S-Trap with 2 and 16 h digestion time, FASP and in solution with 16 h digestion time) methods using 5 replicates of NIST plasma per experimenter (person **A** and **B**) (**B**) Venn diagram showing the total amount of identified proteins of the measurements mentioned in **A**. (**C**) Comparison of the mentioned sample processing procedures in regard to their identified peptides (n = 5)

Mass spectrometric analysis of antibodies in plasma or serum is crucial for understanding immune responses, biomarker-based disease diagnostics, and therapeutic monitoring approaches. The quality of the sample matrix, especially plasma/serum, plays a pivotal role. In this study as an exploratory experiment, three different sample preparation methods – in solution digestion, FASP (Filter-aided Sample Preparation), and S-Trap (Suspension-Trapping) - were comprehensively investigated to determine which method achieves the optimal balance between peptide detection, minimizing missed enzyme cleavage sites during protein digestion, and attaining the highest reproducibility. Analyses of the samples using Orbitrap mass spectrometers in Data-Independent Acquisition (DIA) mode initially focused on the quantitative assessment of the identified proteins (Fig. 3A). Each sample preparation method was conducted 10 times independently. For evaluation and normalization, Spectronaut software was employed. The results clearly indicate that there are only minimal differences in the number of identified proteins between the various processing methods, as illustrated in Fig. 3A. All methods showed a good reproducibility with CVs of 1% (S-Trap 2 h), 3.1% (S-Trap 16 h), 0.9% (FASP) and 1.3% (in solution).

To assess whether a specific method exhibits superior processing capabilities for certain protein groups, the various segments of the Venn diagram (Fig. 3B) were scrutinized. The utilization of the 30 kDa filter in FASP processing hindered the detection of mostly smaller peptides with this method. Apart from this limitation, no discernible groups were identified as being exceptionally well-processed by any of the four methods. We then compared the ratio of missed cleaved peptides (MCP) to fully cleaved peptides (FCP) to evaluate the different processing procedures and to check whether the elevated temperature justifies the short digestion time of 2 h for S-Trap (Fig. 3C). With a MCP proportion of 35%, the short digestion displayed a significantly higher missed-cleavage rate compared to overnight digestion (20% for S-Trap, 23% FASP, and 20% in solution). Consequently, the short digestion time cannot be justified for this type of experiment.

We identified in solution Digestion as the optimal method for processing plasma and serum samples due to its rapid and easily scalable execution, high reproducibility, low number of missed cleavages, and only minor differences in the number of identified proteins. These parameters hold particular significance, especially for clinical applications.

### Testing different trypsin vendors to achieve maximum digest yield

In order to efficiently and precisely detect and quantify antibodies in TDM, it must be ensured that a maximum digestion yield of the molecule is achieved. The quality of the trypsin used has a significant influence on the specificity and efficiency of the hydrolysis. By optimizing these, the formation of non-specific (non-tryptic) peptides and peptides with missing cleavage sites per protein is reduced. As a result, a greater number of unique, fully cleaved tryptic sequences can be identified in mass spectrometry measurements. This enables a more efficient use of resources. In addition, peptides with missing or incorrect cleavage sites are often excluded from protein quantification, emphasizing the need for complete digestion for reliable quantification.

**Fig. 4:**
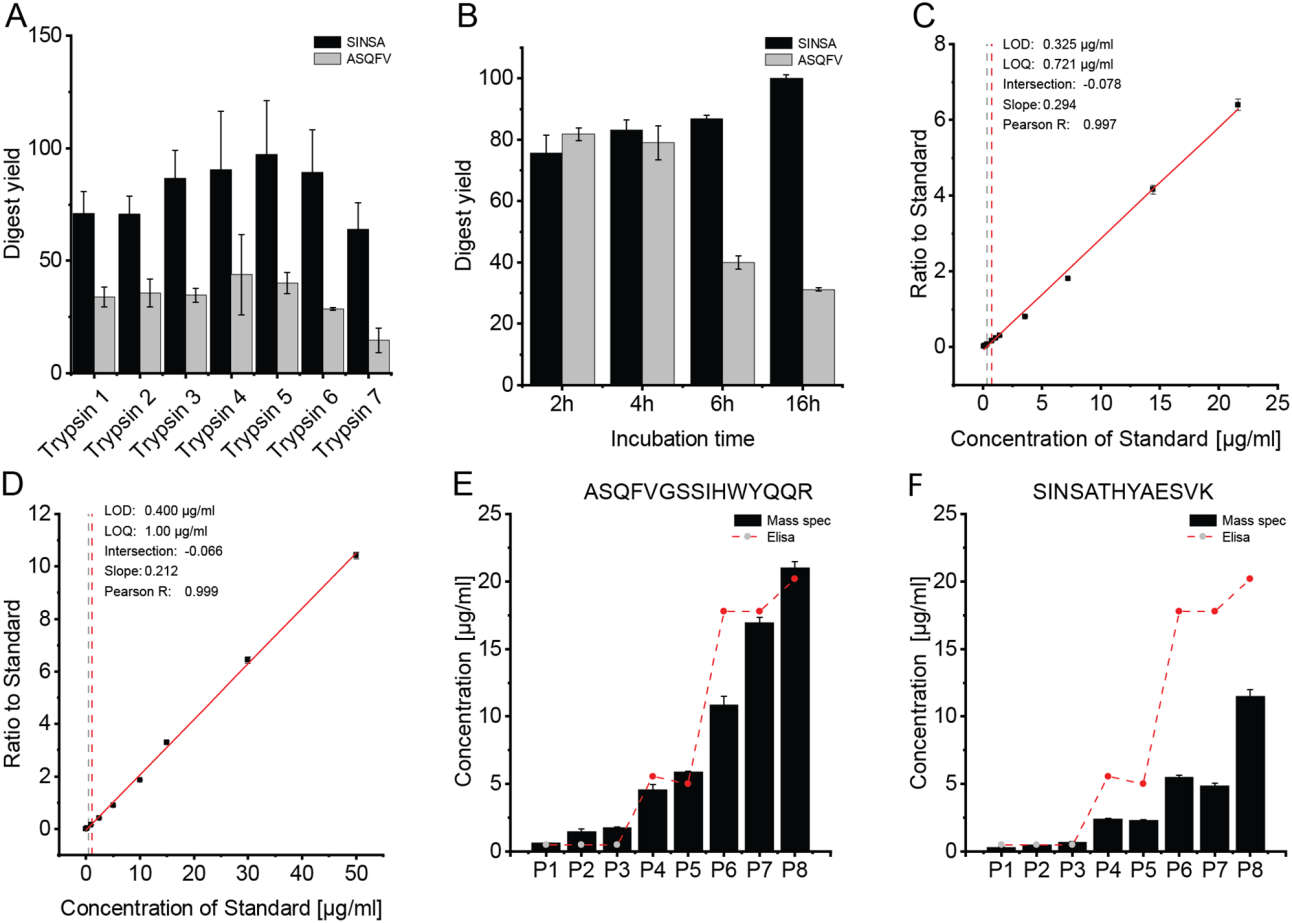
Testing and optimizing enzyme hydrolysis of the antibody: (**A**) Calculated digest yield of both investigated peptides with different trypsins in a plasma sample matrix. (**B**) Digest yield of the peptides of choice after different incubation times for proteolysis. (**C**-**D**) Calibration curves of the SINSA peptide of infliximab. Curves were generated and measured by two different researchers with a time difference of 6 month. Both calibration curves show robust results in regard to their LOD and LLOQ. Each calibration point was measured 3 time with independent replicates. (**E**-**F**) Results of the absolute quantification of the peptides shown in 8 different patient samples (serum). Shown are the antibody concentrations determined by an ELISA (dashed line) and the measured values obtained by mass spectrometry in comparison (black bars). The measurements were carried out on identical samples from each patient

To address this, in a second exploratory experiment various hydrolases (specifically trypsin) from different manufacturers were evaluated in the subsequent experiment. The objective was to determine which trypsin, sourced from different manufacturers, would yield the best results for the previously identified antibody peptides. The digestion yield was then quantified for both the SINSATHYAESVK (SINSA) and ASQFVGSSIHWYQQR (ASQ) peptides. The results (Fig. 4A) indicate that trypsin 5 yielded the most favorable outcomes. With a digestion yield of 40% (CV 4.8%) for the ASQ peptide and 97% (CV 23.9%) for the SINSA peptide, trypsin 5 demonstrated superior efficiency in digestion. While trypsin 4 achieved comparable values, the corresponding standard deviations were considerably larger.

To explore the difference digest efficiencies subsequently, we further investigated and compared different incubation times with the trypsin determined from the preliminary test in order to find out when the yield of generated peptides reaches its maximum. Based on the results, the digestion times can be adjusted to possibly save time during sample preparation.

### Investigation of peptide yield to assure accurate antibody quantification

The choice of optimal digestion time profoundly influences the efficiency and completeness of the enzymatic cleavage process, directly impacting the subsequent identification and quantification of peptides. Especially in the field of TDM, where the precise determination of antibody concentration in the patient is of utmost importance and insufficient, non-reproducible digestion distorts the quantification and can therefore lead to erroneous analysis results, this topic receives significantly greater emphasis. Therefore, the yield of tryptic digestion was determined in a time course experiment by MRM analysis, using the two previously used tryptic peptides of the infliximab antibody as target sequences. The yield of the chosen peptides for the tryptic digestion over time using trypsin 5 is shown in Fig. 4B. Yields of 81.24–99.21% were achieved with the SINSA-peptide. The lowest yields were achieved after 2 h. There was an increase in yields between 2 and 4 h of incubation time. The highest average yield (99.21%) was achieved with trypsin 5, after 16 h of incubation. On the contrary when quantifying the ASQ-peptide, it can be seen that the yields decrease with increasing incubation time for trypsin 5. In contrast to quantification of the SINSA-peptide, the highest yield for the ASQ-peptide (81.83%) was achieved after 2 h and decreased with increasing incubation time (Fig. 4B). It is noticeable that the standard deviation is lowest after 16 h of digestion, especially for the ASQ-peptide, however the difference in the yield of each peptide is best after 2 h of incubation. In pursuit of our primary objective to develop a highly sophisticated and reliable method for quantifying Infliximab, we opted for a 2-hour digestion time. This duration was selected because it demonstrates the most consistent stability in yield for both peptides. For this reason, a digestion time of 2 h is recommended and was used for the further experiments analyzing patient sera.

### Robust calibration curves ensuring accurate quantification for infliximab

For calibration, ascending concentrations or moles of the native or endogenous (NAT) peptide were measured, while the concentration of the stable isotope labeled (SIL) peptide was kept constant at 5 µg/ml or 347.20 fmol throughout all measurements. Normalization was subsequently performed based on the ratio of NAT peptide to SIL standard. A calibration curve for both peptides was then generated from the ratio between NAT and SIL peptides. The limit of detection (LOD) was calculated by adding the blank with three times the standard deviation. The lower limit of quantification (LLOQ) for quantification was defined as the smallest point on the calibration curve with a maximum error and coefficient of variation of 20%. Figure 4C illustrates the calibration curve for the SINSA-peptide. The equation of the curve is determined by the slope (m) and the y-intercept (b) using the linear equation 𝑦=𝑚𝑥+𝑏. Applying this formula, the equation for is 𝑦=0.2120𝑥−8.0406 × 10 − 2. The coefficient of determination (R²) is 0.9985. Using Skyline, the LOD was calculated to be a concentration of 0.4006 µg/ml, and the LLOQ was determined to be a concentration of 1 µg/ml. The presented calibration curve for the ASQ-peptide (see supplement Figure S1) can be described by the linear equation 𝑦= 0.16845𝑥−3.2639 × 10 − 2 and has an R² of 0.9992. For the limit of detection, a concentration of 0.3121 µg/ml was calculated, and the limit of quantification, as with Peptide 1, is 1 µg/ml.

To assess the robustness of our method in terms of precision and accuracy using NIST plasma and mAB spike-in analyses, particularly concerning the generation of calibration curves for peptides of infliximab and the associated derivation of LOD and LLOQ, two researchers were assigned the task of independently preparing and measuring the calibration curves of the SINSA peptide of infliximab. This was done with a time interval of 6 months. The LOD of the mAB exhibited a consistently narrow range of 0.3 to 0.4 µg/ml for both calibration curves. Similarly, the LLOQ showed a tight range of 0.7 to 1 µg/ml (Fig. 4C-D).

### Evaluating therapeutic antibody levels with UHPLC-MRM-MS and ELISA

In this manuscript, we demonstrate the highly reproducible detection and quantification of single mAB by UHPLC-MRM-MS with stable isotope-labeled peptides. The established SOP using a final digestion time of 2 h shows excellent precision and accuracy within the therapeutic range of therapeutic antibodies. The data obtained from a small cohort of patients are compared with the results of a routine ELISA measurement for the identical samples, which were also analyzed by UPLC-MS. In this way, this assay provides the potential to identify patients who produce antibodies that interfere with the ELISA-based detection and therefore provide too low values for the serum levels of the therapeutic antibodies.

Figure 4E-F depicts the absolute quantification of the two peptides in 8 different patient serum samples. In addition, the antibody concentrations determined by ELISA (dashed line) are shown for comparison. The measurements were carried out on identical samples from each patient.

It is noticeable that although the values of both peptides correspond to the ELISA values in terms of trend, the values of the peptide ASQFVGSSIHWYQQR are closer to those of the ELISA. The latter were only determined by single point measurements.

In addition, both infliximab peptides could not provide results for one sample, as the concentration was below the quantification limit in both cases, which is also consistent with the ELISA data. However, concentrations could be calculated for all other samples. The range of calculated values ranged from 0.65 µg/ml (± 0.01 µg/ml) infliximab to 21.00 µg/ml (± 0.38 µg/ml) for the peptide ASQFVGSSIHWYQQR while the values for the peptide SINSATHYAESVK were calculated at 0.31 µg/ml (± 0.01 µg/ml) to 11.50 µg/ml (± 0.44 µg/ml).

In all samples, it can be clearly seen that the infliximab concentrations determined in the patient samples differ significantly from peptide to peptide. When calculating the concentration with the peptide ASQFVGSSIHWYQQR, the concentrations determined are about twice as high in most cases compared to the peptide SINSATHYAESVK. Theoretically, the calculated infliximab concentration in the patient samples should result in the same values for both peptides. The protein concentrations were not adjusted for the observed differences in quantifications. These variations are likely attributable to multiple factors rather than a single cause. Possible underlying reasons may include differences in the accessibility of peptides for proteolytic cleavage by trypsin. The light chain, encompassing the hypervariable region, must be readily accessible to other proteins for functional fulfilment, whereas the Fc-part of the antibody may not be as accessible to the protease. For instance, the N-terminal region of the selected peptide, located in a beta-strand, may not be as readily cleaved as peptides from less structured regions.

Moreover, post-translational modifications, although expected to have a negligible impact on quantifications, were not considered in this analysis. However, deamidation of the heavy chain peptide has been demonstrated to occur, albeit to a very minor degree [15, 16]. Additionally, a slight oxidation of the light chain peptide was observed during our study, but to a minor extent.

Given that blood sampling and the duration until freezing in hospitals are typically challenging to control, these modifications could occur within the first minutes to hours before sample preparation, potentially contributing to alterations in quantifications in real-life samples. Moreover, unidentified factors may also contribute to this effect.

It should be emphasized that the ELISA values for two additional patients were below the detection limit, while the MRM measurements were able to provide concentration levels.

## Discussion

Accurate and vigilant monitoring of antibody concentration levels in patients undergoing targeted drug therapy is paramount for optimizing treatment outcomes and ensuring patient safety. This practice is particularly crucial due to several interconnected factors that influence the therapeutic effectiveness and safety of drugs. Personalized medicine relies on the understanding that individuals metabolize drugs differently. Monitoring antibody concentrations allows healthcare providers to tailor treatment plans to the unique characteristics of each patient, accounting for factors such as age, weight, and specific health conditions. This individualized approach not only enhances therapeutic efficacy but also minimizes the risk of adverse effects associated with either suboptimal or excessive drug levels. Maintaining drug concentrations within the therapeutic range is essential to achieving the desired therapeutic effects while minimizing side effects. By avoiding concentrations below the therapeutic range, practitioners ensure that the drug remains effective. Simultaneously, monitoring prevents concentrations above the therapeutic range, mitigating the risk of adverse reactions and toxicity.

An obstacle encountered in the here employed targeted MS approach involves the differentiation of the proteotypic infliximab peptides from the diverse array of endogenous immunoglobulins found in human serum. To enhance specificity, the monitoring strategy incorporates two peptides, sourced from both the heavy chain and the light chain of infliximab. In addition, both peptides were chosen as they are part of the hypervariable and interaction regions of the antibody.

In clinical practice, infliximab is considered undetectable if its concentration in the bloodstream is below 1.0 µg/ml [17]. Medical decisions to adjust the dose or infusion intervals are usually based on infliximab measurements at trough levels below 3–5 µg/ml. In addition, the wide dynamic range of the assay established here, allows for the measurement of high infliximab concentrations up to 50 µg/ml. Although the MRM method has a higher limit of quantification (LLOQ) compared to commercially available ELISA tests, it was able to successfully quantify and differentiate clinically significant concentrations of infliximab in serum. We have closely matched the LLOQ of assays currently available on the market.

A notable advantage of the presented semi-automated sample preparation in combination with LC-MS/MS analysis are the high and consistently reproducible results achieved by automated and standardized processes. This workflow makes it possible to obtain results for more than 90 patient samples within 2 days. While many laboratories have established semi-automated procedures for ELISA that allow for faster measurements, these methods can only detect a single protein per assay. In contrast, targeted mass spectrometry methods can detect multiple proteins simultaneously in a single sample, facilitating the direct inclusion of potentially interfering anti-drug antibodies (ADAs). In addition, samples from patients undergoing combination therapy with multiple antibodies can be detected in a single measurement. The assays developed are therefore highly scalable. Targeted peptide measurements directly in the variable region of the antibody avoid false-positive results due to cross-reactivity, enabling more precise dosing options for individual patients and advancing precision medicine.

With the presented setup, we were able to demonstrate the stability of the established calibration curves for specific infliximab peptides over a longer period of time, even if they were generated by different persons. This indicates the reliability and robustness of the values obtained. However, another challenge is the labor-intensive assay development, which requires careful selection and validation of peptides for each antibody to be tested. This process should take into account the antibody regions, digestion efficiency during sample preparation and key performance indicators such as LOD and LLOQ, as well as reproducibility, which can be affected by degradation effects and oxidation of certain amino acids. A minor drawback of the study conducted is the limited number of patients, although the inclusion of ELISA values increases its validity.

## Conclusions

In conclusion, our study demonstrated the detectability and quantifiability of infliximab in the serum of treated patients using LC-MS/MS and variable region proteotypic peptides. Therapeutic antibodies such as infliximab are a costly treatment option and the use of laboratory data to assess treatment response could improve patient care while significantly reducing healthcare costs. This cost-effective analytical approach has the potential for rapid adaptation to other monoclonal antibody therapeutics. It also paves the way for future clinical studies to determine associations with prognosis and outcomes of various autoimmune diseases for which TNF inhibitors, including this one, are commonly prescribed.

### Electronic supplementary material

Below is the link to the electronic supplementary material.

**Supplementary Material 1:** Calibration curves of the ASQ peptide of infliximab. Curves were generated and measured by two different researchers with a time difference of 6 month. Both calibration curves show robust results in regard to their LOD and LLOQ. Each calibration point was measured 3 time with independent replicates. The LOD is marked with the purple line, the LLOQ is marked with the blue line

**Supplementary Material 2:** Overview of missed cleaved sites for each sample preparation method. (FCP= fully cleaved peptide, MCP= missed cleaved peptide)

## Data Availability

The datasets used and/or analysed during the current study are available from the corresponding author on reasonable request.

## Footnotes

1 Zentrale Ethikkommission der Bundesärztekammer. Dtsch Arztebl 2003; 100: A 1632.

## Abbreviations

TDM: target drug monitoring
ELISA: Enzyme-Linked Immunosorbent Assay
IBD: inflammatory bowel disease
MAbs: monoclonal antibodies
IFX: infliximab
ADM: Adalimumab
TNF-α: Tumor necrosis factor alpha
TCEP: Tris(2-carboxyethyl) phosphine hydrochloride
IAA: Iodoacetamide
DTT: dithiothreitol
TFA: trifluoroacetic acid
FA: formic acid
TEAB: triethylammonium bicarbonate
ABC: ammonium bicarbonate
FASP: Filter-aided Sample Preparation
DIA: Data independent acquisition
MCP: missed cleaved peptides
FCP: fully cleaved peptides
