# Supplementary material for: Highly sensitive therapeutic drug monitoring of infliximab in serum by targeted mass spectrometry in comparison to ELISA data
Source: Clin Proteomics. 2024 Feb 29;21:16. doi: 10.1186/s12014-024-09464-x (PMC10905900; doi:10.1186/s12014-024-09464-x)

**Supplementary information**

**Highly sensitive therapeutic drug monitoring of infliximab in serum by targeted mass spectrometry and ELISA**

Andreas Hentschel, Gina Piontek, Rob Dahlmann, Peter Findeisen, Roman Sakson, Phil Carbow, Thomas Renné, Yvonne Reinders*, Albert Sickmann*

*corresponding author

Supplementary Figure S1: Calibration curves of the ASQ peptide of infliximab. Curves were generated and measured by two different researchers with a time difference of 6 month. Both calibration curves show robust results in regard to their LOD and LLOQ. Each calibration point was measured 3 time with independent replicates. The LOD is marked with the purple line, the LLOQ is marked with the blue line.


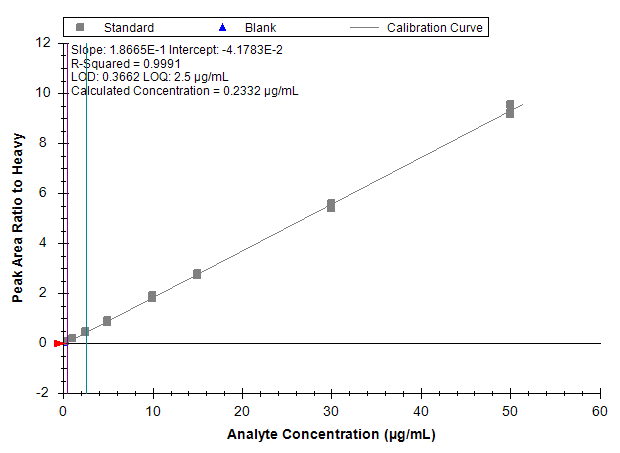

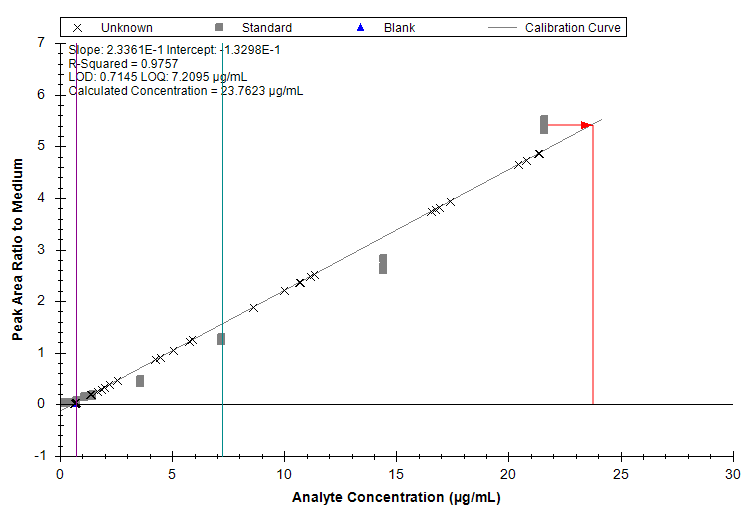

Supplement: Supplementary file 1 — Supplementary Material 1: Calibration curves of the ASQ peptide of infliximab. Curves were generated and measured by two different researchers with a time difference of 6 month. Both calibration curves show robust results in regard to their LOD and LLOQ. Each calibration point was measured 3 time with independent replicates. The LOD is marked with the purple line, the LLOQ is marked with the blue line [file 12014_2024_9464_MOESM1_ESM.docx]
