# Supplementary figures and images for: Highly sensitive therapeutic drug monitoring of infliximab in serum by targeted mass spectrometry in comparison to ELISA data
Source: Clin Proteomics. 2024 Feb 29;21:16. doi: 10.1186/s12014-024-09464-x (PMC10905900; doi:10.1186/s12014-024-09464-x)

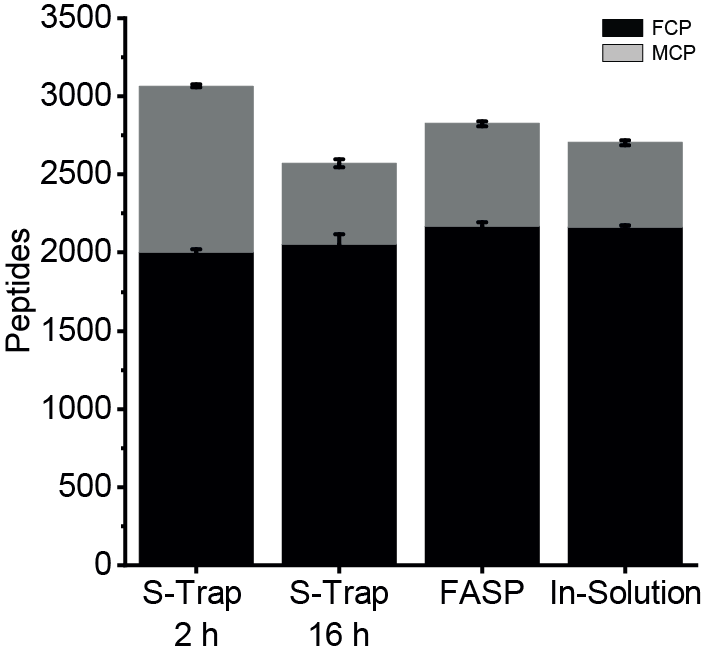

Supplement: Supplementary file 2 — Supplementary Material 2: Overview of missed cleaved sites for each sample preparation method. (FCP= fully cleaved peptide, MCP= missed cleaved peptide) [file 12014_2024_9464_MOESM2_ESM.png]
